# Electrical Stimulation for Stem Cell-Based Neural Repair: Zapping the Field to Action

**DOI:** 10.1523/ENEURO.0183-24.2024

**Published:** 2024-09-06

**Authors:** Stephanie N. Iwasa, Xilin Liu, Hani E. Naguib, Suneil K. Kalia, Milos R. Popovic, Cindi M. Morshead

**Affiliations:** ^1^The KITE Research Institute, Toronto Rehabilitation Institute—University Health Network, Toronto, Ontario M5G 2A2, Canada; ^2^CRANIA, University Health Network and University of Toronto, Toronto, Ontario M5G 2A2, Canada; ^3^Department of Electrical and Computer Engineering, University of Toronto, Toronto, Ontario M5S 3G4, Canada; ^4^Department of Mechanical and Industrial Engineering, University of Toronto, Toronto, Ontario M5S 3G8, Canada; ^5^Institute of Biomedical Engineering, University of Toronto, Toronto, Ontario M5S 3G9, Canada; ^6^Department of Materials Science & Engineering, University of Toronto, Toronto, Ontario M5S 3E4, Canada; ^7^Department of Neurosurgery, University Health Network, University of Toronto, Toronto, Ontario M5T 2S8, Canada; ^8^Krembil Research Institute, Toronto, Ontario M5T 2S8, Canada; ^9^Department of Surgery, University of Toronto, Toronto, Ontario M5T 1P5, Canada

## Abstract

**Figure:**
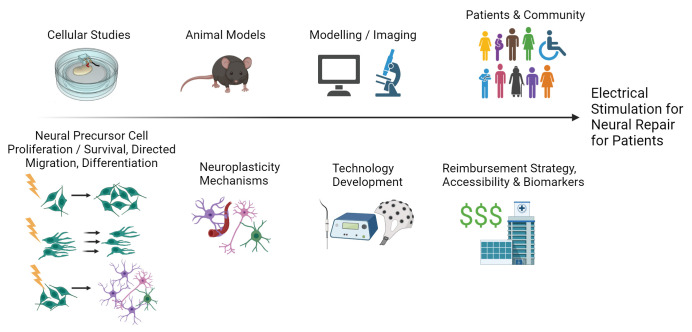
Visual Abstract

Visual Abstract

## Significance Statement

A multidisciplinary and international convergent working group (neural stem cell biology, functional electrical stimulation, materials engineering, electrical engineering, neurosurgery, neurology, biomedical device, and commercialization) met in Canada to create a call to action for Electrical Stimulation for Neural Repair. Electrical stimulation, in the form of deep brain stimulation (DBS), is an approved treatment for various neurological disorders such as essential tremor, Parkinson's disease, and epilepsy. Here, DBS works through disrupting neural circuits; however, electrical stimulation may also effectively promote neural repair due to the activation of electrosensitive resident neural stem cells. Activating neural stem cells has great promise for enhancing neuroplasticity to treat damaged brains. To realize this therapy's potential, multidisciplinary experts met to identify barriers, gaps, and next steps.

## Introduction

Neurological disorders are a leading cause of death and disabilities worldwide and represent an enormous public health challenge (Feigin and Vos, 2019). A conservative estimate of the global financial burden is more than two trillion US dollars/year, and these costs are expected to double by 2030. For those afflicted, they have a decreased quality of life, and their dependence on others places a heavy burden on our society and healthcare networks. The major neurological disorders contributing to the overall burden are stroke and dementias (including Alzheimer's disease; Feigin et al., 2020). There are presently no cures and limited treatment options for stroke or neurodegenerative diseases. There is a clear need and incentive for therapeutic interventions to promote neural repair and functional recovery.

Neural stem cells and their progeny (together termed neural precursor cells, NPCs) reside within well-delineated niches in the central nervous system (CNS). These resident cells are self-renewing and can differentiate into the different cell types of the brain, which has spurred interest in the design of strategies to harness NPC potential for promoting neural repair (Reynolds and Weiss, 1992; Iwasa et al., 2020; Purvis et al., 2020). Modulating NPC behavior has been correlated to functional recovery in models of brain injury using drugs and small molecules (R. L. Zhang et al., 2006, 2012; Sachewsky et al., 2014; Tang et al., 2023). NPCs are electrosensitive cells affording the opportunity to use electrical stimulation as a prospective repair strategy (Ariza et al., 2010). Electrical stimulation is a compelling treatment option because of the spatial and temporal precision afforded by its application, particularly when applied invasively. Furthermore, electric fields are also present endogenously and are important during development and wound healing. These endogenous fields influence NPC behavior within the mature CNS such as migration along the rostral migratory stream and in the corpus callosum (Cao et al., 2013; Iwasa et al., 2019). Electric field application can increase NPC proliferation, direct migration, and modulate differentiation (Babona-Pilipos et al., 2011; Chang et al., 2011).

Electrical stimulation is widely used as a therapeutic treatment. In deep brain stimulation (DBS), implanted electrodes commonly deliver electrical stimulation to targeted regions such as the basal ganglia and thalamus to treat movement disorders, epilepsy, and pain. New indications for DBS such as obsessive–compulsive disorder, depression, Alzheimer's disease, and obesity are emerging (Lozano et al., 2019; Jakobs et al., 2020; Hitti et al., 2023). Electrical stimulation to the brain can also be delivered noninvasively through transcranial electrical stimulation to treat neurological disorders (e.g., depression; Bhattacharya et al., 2022). Electrical stimulation is delivered to the spinal cord for pain and to peripheral nerves (termed functional electrical stimulation, FES) for rehabilitation after stroke or spinal cord injury. These forms for electrical stimulation work and the parameters (duration, intensity) vary depending on the disease/injury. We propose that electrical stimulation can be a novel, safe, and effective strategy to activate resident NPCs and lead to neural regeneration and improved functional outcomes.

To bring an electrical stimulation-mediated treatment option for neural repair from cells to patients, a multidisciplinary approach is necessary. The mechanism of NPC activation needs to be elucidated to determine the appropriate stimulation paradigm. Electrodes and stimulators for animal model testing and with specifications for NPC activation need to be designed. Furthermore, the technologies and stimulation paradigms need to be designed with patient needs, usability, and marketability in mind. This would involve considerations of benefits and limitations of invasive and noninvasive stimulation as well as using wearable technologies. To address these prerequisites, multidisciplinary experts met for a Mini-Symposium and Workshop held in February 2023 in Canada to establish the state of the field, challenges, and directions. All participants were volunteers and included internationally recognized experts in their respective fields. There were early career investigators and patient-facing practitioners within and outside of Canada. Here, we aim to generate excitement for the field while presenting relevant background and illustrating the main challenges and next steps to bringing electrical stimulation for neural repair as a treatment to fruition.

## Mechanism of Neural Repair Using Electrical Stimulation to Activate Neural Stem Cells

Neural stem cells are found during development, adulthood, and throughout aging (Negredo et al., 2020). These self-renewing, multipotent cells can be isolated from along the entire neuraxis, where they are found in highly specified and restricted niches including the periventricular region lining the ventricles. In the brain, NPCs are migratory cells that proliferate and contribute to olfactory bulb neurogenesis. In vitro, individual neural stem cells give rise to NPC colonies of cells, termed neurospheres. This forms the basis of the simple and robust neurosphere assay used to assess the size of the NPC pool. Following injury alone, NPCs can be “activated,” meaning there is an increase in neural stem cell proliferation, survival, migration, and neurogenesis (Arvidsson et al., 2002; Li et al., 2010; Faiz et al., 2015), albeit limited. While this injury-induced activation is not sufficient for neural regeneration and functional recovery, the administration of small molecules, drugs, and growth factors to enhance NPC activation is correlated with improved tissue and functional outcomes (C. Zhang et al., 2010; Jeffers et al., 2014; Dadwal et al., 2015). We look to enhance resident NPC activation using electrical stimulation. Indeed, electrical stimulation has been shown to increase NPC proliferation, direct migration, and modulate differentiation both in vitro and in vivo in rodents (Feng et al., 2017; Sefton et al., 2020).

Studies have also shown that electrical stimulation can lead to functional recovery and neurogenesis (Balseanu et al., 2020). The correlation between functional benefit and NPC activation in a disease model has been demonstrated in a number of settings (Dadwal et al., 2015; Battistini et al., 2023; Gilbert et al., 2023), and knock-out models of neural stem cells have provided evidence to support the relationship between neural stem cell activation and improved outcomes (Williamson et al., 2023). Indeed, work is still needed to solidify the direct link between neural stem cell activation via electrical stimulation and functional recovery, and rigorous preclinical injury model experiments required for these studies are ongoing.

Ongoing studies that deliver electrical stimulation after an injury (e.g., stroke) and compare with injured mice with implants and no stimulation can establish the effectiveness of the treatment using behavior tasks before and after injury. Once functional recovery is observed, NPC kinetics (proliferation, survival, migration, and differentiation into mature neural cells) can be examined using in vitro, ex vivo, in vivo, and in silico methodologies (Meng et al., 2011; Sefton et al., 2020; Zimmerman et al., 2021). Two aspects of these studies are critical for interpretation. First, it is important to perform these studies in transgenic mouse models that enable lineage tracking of endogenous NPCs (Albright et al., 2016). Labeling the endogenous NPCs prior to injury will confirm that electrical stimulation of the NPCs leads to their activation and contribution to tissue repair. Important studies employing inducible NPC ablation in transgenic mice followed by electrical stimulation will be needed to definitively demonstrate that NPC activation is necessary and/or sufficient for the recovery.

The stimulation parameters (strength, frequency, duration, waveform shape) and the brain region stimulated can affect the extent of NPC migration and proliferation (Lau et al., 2023). Optimized parameters and the timing of the intervention after injury or during a disease progression are also critical factors for successful treatments (Dromerick et al., 2021). Additionally, observation on the effect of age and sex on NPC response to electrical stimulation is important as environmental factors affect NPC response as well (Ahmed et al., 2020).

Concomitant with optimizing NPC activation, our studies need to address fundamental biological questions to understand how the electrical stimulation-mediated repair is affecting the cells and circuitry to support neuroplasticity. For example, DBS has been demonstrated to disrupt neural circuits (Milosevic et al., 2018); how will the stimulation designed for NPC activation affect aspects of neuroplasticity such as axon regeneration, synaptic connection, modified neural circuits, glial cells, and changes in brain vasculature or the blood–brain barrier? Technical and experimental expertise in wet labs using animal studies, state-of-the-art imaging, and recording technologies and modeling can help elucidate the answers to these questions.

## Developing Electrical Stimulation Devices and Therapies for Neuroregeneration

Research labs and clinics regularly use stimulators and electrodes for DBS, transcranial stimulation, and transcutaneous stimulation as well as employ electrodes for recordings. The current tools are not customized for examining and optimizing the effects of electrical stimulation on NPCs to investigate the questions posed above. Multidisciplinary efforts to acquire an understanding of the limitations of stimulators (electrical engineering) and electrodes (device design/materials engineering) is needed alongside the development of intervention. There are several main considerations/challenges with respect to stimulator and electrode design, development, and fabrication: (1) safety, generating charge-balanced stimulation that does not cause tissue damage over long-term, continuous stimulation delivery; (2) performance, generating the waveform with optimized parameters and delivering these in real-world settings with noise, varying tissue contact impedances, and other confounders; (3) determining electrode placement and the number of the electrodes needed to deliver electrical stimulation most conducive to activating endogenous NPCs; (4) shape and insulation of the electrodes to produce desirable electric fields in vivo; (5) miniaturization, allowing devices to be used in small animal models during freely behaving experiments and eventually enable the translation to implantable and potentially wearable devices; and (6) powering, having a safe, reliable, and lightweight power source that can remain in or on the body for prolonged periods of time without causing damage to the surrounding tissue (e.g., wireless power systems or battery housing systems that prevent leakage) that is critical (de Haas et al., 2012; Shepherd et al., 2018; Alpaugh et al., 2019). Validation experiments are essential for testing the technology in preclinical animal models prior to human application to determine whether these considerations have been addressed appropriately (e.g., consistent results in activating NPCs, no changes in behavior while wearing the stimulator, no tissue damage).

For NPC activation, the implanted electrodes need to stimulate the endogenous neural stem cell niche, which comprises the periventricular region of the forebrain. These stimulating electrodes could resemble DBS electrodes; hence considering existing DBS electrodes designs, stimulation patterns, and postmortem tissue responses is relevant (Cagnan et al., 2019; Evers and Lowery, 2021). However, certain characteristics will likely need adjustment for NPC activation. For example, current treatment of progressive diseases such as Parkinson's disease dictates ongoing/continuous stimulation (although adaptive DBS is being investigated to consider how a patient's symptoms and needs can fluctuate throughout the day; van Wijk et al., 2023). It may be different in injury models, such as stroke, where a permanent implant may not be required. One could envision implantation of the device early after injury with delivery of the stimulus while the patient is receiving physical and occupational therapy treatments or implantation in the subacute or chronic phases poststroke to enhance NPC activation (Boese et al., 2018). In injury models that are not progressive diseases, it may serve to remove the stimulator and the electrode. In that embodiment of the stimulation technology, it may be beneficial to have electrodes and cabling that degrades over time to allow for the effective removal of the implanted device (Shim et al., 2021). Alternatively, stimulation could be applied noninvasively. This would solve the challenge of removing the stimulator and electrodes; however, modeling and analysis would be required to accurately determine spatial and temporal control of the electrical stimulation in targeted regions. Stroke represents an enormous clinical population making these considerations important engineering goals for system design and implementation.

To enhance repair in various neurological disorders, electrical stimulation can be provided in combination with drug delivery, cell transplantation, and/or rehabilitation. Drug delivery and/or cell transplantation along with electrical stimulation could be achieved through multimodal electrodes. For example, electrodes could be designed to deliver electrical stimulation and provide immunomodulatory and/or neuroprotective compounds through controlled release of drugs such as brain-derived neurotrophic factor and short interfering RNAs (Iwasa et al., 2020; Barman and Jhunjhunwala, 2024). Alternatively, or in addition, electrical stimulation could be combined with NPC transplantation (which has been used in clinical trials and shown promise; Leone et al., 2023). Electrical stimulation may improve functional recovery by improving cell survival which is invariably low following transplantation (Tejeda et al., 2022; Leone et al., 2023). Electrical stimulation could be delivered to the brain in conjunction with rehabilitation treatments to support neuroplasticity, integration of surviving cells in the neurocircuitry, and/or cell survival (Corbett et al., 2015). This potential is highlighted by studies in the peripheral nervous system, whereby FES combined with rehabilitation leads to functional improvements through neuroplasticity (Marquez-Chin and Popovic, 2020). Many electrical stimulation combinatorial strategies are possible, and synergies between these strategies may augment the extent of recovery for individuals with neurological disorders.

## Clinical Relevance—Biomarkers, Therapy Adoption, and Reimbursement

To test the success of the therapy and adjust the therapy for human application, we need relevant biomarkers as measures of success. In preclinical models, behavioral outcomes and brain tissue analyses are used to evaluate success, but in humans the outcome measures are more limited. Potential biomarkers could be related to brain imaging (e.g., diffusion tensor imaging for corticospinal tract analysis; Findlater et al., 2019; Moura et al., 2019), neural recordings (e.g., local field potential changes similar to what is used for adaptive DBS; Vissani et al., 2020), electromyography recordings (e.g., changes in muscle synergies; Irastorza-Landa et al., 2021), or motion sensors (e.g., changes in ranges of motion; Derungs et al., 2020). As digital health technologies such as wearable devices become more common, they can serve as an accessible alternative to record data and aid in the identification of functional and sensitive digital biomarkers, ultimately guiding the optimization of the stimulation parameters (i.e., timing and dosing) of the therapy (Pathak et al., 2021). If a technology is wearable, it may be easier for underserved populations (e.g., remote communities, previously discriminated against communities) to access and participate in therapy development. It is important to have a diverse group of end-users participate in the process as each will have usability suggestions and different needs (Levine et al., 2020; Adler et al., 2023), leading to improved therapy relevance for a greater number of patients.

To bring this therapy from bench to bedside, all aspects of the therapy implementation need to be considered, and above all, the therapy adoption and reimbursement strategies need to be considered early in the development phase. Efforts to demonstrate that this therapy modality produces significant and clinically meaningful impact on patients and their family members are the most clinically relevant outcomes; however, the cost-effectiveness of continuing with the present treatment modalities versus this proposed intervention must be carefully assessed. If a clear business model shows functional improvements for patients and captures savings for a specific territory's healthcare system, then discussions and negotiation around reimbursement in targeted regions for technology deployment are warranted (Stieglitz, 2020; Smuck et al., 2021). A therapy is only worth pursuing if it positively impacts patient outcomes. Therefore, the research team has to engage regulatory, ethics, and business people as soon as the therapy shows clinical promise.

## Next steps

The use of electrical stimulation to activate NPCs with the objective of developing next-generation neuroregenerative treatments and technologies is very attractive. Electrical stimulation has been used for over six decades in rehabilitation, in cardiac pacemakers and hearing aids, and more recently for DBS systems. This is a mature technology that can be relatively easily adopted to promote neuroregenerative processes based on resident NPC activation. However, even though this technology is mature and available, it will require considerable effort from various disciplines to make it relevant and appropriate for injury/disease applications. We will need neurobiologists and stem cell biologists, neural engineers, material and manufacturing experts, electrical engineers, data scientists, neurosurgeons, neurologists, regulatory experts, and commercialization experts to move this field forward. We will also need the patient, caregiver, and community groups to define the unmet need as well. Each group brings their unique perspective, expertise, and ideas and can identify the promise and the gaps that need to be addressed before this technology becomes a reality. We identified the multidisciplinary team of people to bring to the conversation and engage in meaningful discussion (Table 1).

**Table 1.** Research areas to target and people to consult to develop electrical stimulation for neural repair

Research areas to target	People to bring to the conversation
Understanding the mechanisms underlying the proposed therapies to adapt and optimize the therapy Technology development to go from cell-level experiments to patient implementation through unified research objectives and goals (e.g., stimulation/device to activate NPCs, devices/technology already available and modifications to move the field forward, and validation methods to demonstrate technology efficacy) Above all, clinical relevance and the therapy's true impact on patients determine therapeutic viability. We need to determine the value added of the therapies/devices once they are delivered to the patients, accessibility of the therapies for patients, reimbursement strategies, and patient selection to ensure therapy efficacy	Funding agencies, policymakers, and the public are required to understand and discuss the impact of the proposed technologies and to develop ethical framework fitting the proposed treatment's needs. The same parties need a strategy to support this type of systems research (e.g., design of accessories, connectors, electrodes, batteries, and stimulator housing) instead of glorifying and only funding projects perceived as “cutting edge.” In some instances, small changes to existing hardware and software solutions may be sufficient but will still require time, effort, and resources The scientists’ home institutions need to work with the scientists to provide effective and fast research ethics board review processes, ability to process and protect intellectual property without slowing down the research activity, engage the potential licensee of the technology early in the game, and encourage private–public partnership in technology development Individuals from the lived community are needed to better understand the needs of patients and patient support groups (e.g., emotional impact of leaving family for treatment), as well as additional racial and cultural representation

To address the challenges, we need to establish a hub to educate the community and attract new public and private partners to engage in developing electrical stimulation tools for neuroregeneration. The hub will promote collaborations within themes and across disciplines to engage and learn to speak each other's languages, build teams, connect with physician–scientists, and ensure research endeavors address relevant questions and challenges. This would include a mix of local, regional, national, and international members. The knowledge and strategies from different regions would be helpful in referencing and considering potential healthcare strategies, for example. The established hub can address our long-term call to action (>5 years):

1. Bring an electrical stimulation regenerative medicine therapy from cells to patients and provide a model for broad application to diverse neural disorders.
2. Understand the cellular response and mechanism to electrical stimulation for NPC activation through high-resolution imaging technologies and in vitro, ex vivo, and in vivo methodologies.
3. Identify appropriate target biomarkers to more accurately select stimulation parameters and device configurations (hardware and software) and enable the monitoring of patient improvements.
4. Enable long-term monitoring of the patients, their devices, and biomarkers, as a means to enable continuous system and patient experience improvement with the device and the therapy. For example, the stimulation process could be controlled in a closed-loop configuration in real time to accelerate recovery and minimize side effects.
5. Develop standardized and interchangeable electrical stimulators (e.g., open stimulator platforms that could be used off-label for a range of different research uses), electrodes, and monitoring devices for the neural repair field allowing faster deployment of this regenerative medicine technology to patient populations. This will allow for the creation of a neuromodulation “hub” using similar and compatible tools benefiting diverse clinical populations and facilitating rapid interdisciplinary partnerships, knowledge exchange, technology development, and safe clinical deployment.

We believe that the use of electrical stimulation as a means to engage resident NPCs with the objective of developing next-generation neuromodulation treatments and technologies is the future of neuroregenerative medicine. This position paper can be leveraged with policymakers, regulators, funding agencies, and the public. We are looking for like-minded people in academia, industry, healthcare, NGOs, and government to help us move this field forward with the goal making these technologies available to our patients within the next 5–10 years.

## Synthesis

Reviewing Editor: Jennifer Dulin, Texas A&M University Decisions are customarily a result of the Reviewing Editor and the peer reviewers coming together and discussing their recommendations until a consensus is reached. When revisions are invited, a fact-based synthesis statement explaining their decision and outlining what is needed to prepare a revision will be listed below. The following reviewer(s) agreed to reveal their identity: Matthew Hogan.

Reviewer #1:

In this opinion piece, the authors highlight critical thrust areas for the advancement of electrical stimulation targeting nascent neural stem cells as a potential therapy for central nervous system disorders. The outlined approach may have relevance to multiple disorders and impact a wide population should it prove effective.

Critically, there is little information on the overall effectiveness of the strategy itself. The authors highlight that it is necessary to understand whether electrical stimulation is a valid strategy for improving function, however this unmet need is limited to a single sentence. In the opinion of the reviewer, the most critical barrier to advancing the field is a direct demonstration of functional benefit in a preclinical disease model. While two studies demonstrated a correlation between functional benefit and electrical stimulation in a disease model, it is critical to link the "activation" of nascent stem cells to a functional benefit directly. The authors should expand on this unmet need and highlight strategies to address necessary rigorous gain and loss of function experiments.

Further, there is a missed opportunity to address cell-replacement therapeutic strategies. Lessons learned in the context of nascent stem cell activation may reveal potential for synergy/cross-pollination with stem cell graft-based interventions and vice-versa.

In general, the call-to-action outlines major sticking points in advancing the outlined therapeutic strategy, however it fails to highlight the importance of rigor. More discussion of essential and missing pre-clinical modeling experiments is necessary. Still, the authors nicely describe other hurdles necessary to advance this therapeutic approach and present an informative wholistic summary.
